# A Review on Reinforcement Methods for Polymeric Materials Processed Using Fused Filament Fabrication (FFF)

**DOI:** 10.3390/polym13224022

**Published:** 2021-11-20

**Authors:** Juan Pratama, Sukmaji I. Cahyono, Suyitno Suyitno, Muhammad A. Muflikhun, Urip A. Salim, Muslim Mahardika, Budi Arifvianto

**Affiliations:** 1 Department of Mechanical and Industrial Engineering, Faculty of Engineering, Universitas Gadjah Mada, Jl. Grafika 2, Yogyakarta 55281, Indonesia; juan.pratama@mail.ugm.ac.id (J.P.); sukmaji@mail.ugm.ac.id (S.I.C.); akhsin.muflikhun@ugm.ac.id (M.A.M.); urip-as@ugm.ac.id (U.A.S.); 2 Centre for Innovation of Medical Equipments and Devices (CIMEDs), Faculty of Engineering, Universitas Gadjah Mada, Jl. Teknika Utara, Yogyakarta 55281, Indonesia; suyitno@untidar.ac.id; 3 Department of Mechanical Engineering, Tidar University, Jl. Kapten Suparman 39, Magelang 56116, Indonesia

**Keywords:** fused filament fabrication, rapid prototyping, mechanical properties, reinforcement methods

## Abstract

Over the last few years, fused filament fabrication (FFF) has become one of the most promising and widely used techniques for the rapid prototyping process. A number of studies have also shown the possibility of FFF being used for the fabrication of functional products, such as biomedical implants and automotive components. However, the poor mechanical properties possessed by FFF-processed products are considered one of the major shortcomings of this technique. Over the last decade, many researchers have attempted to improve the mechanical properties of FFF-processed products using several strategies—for instance, by applying the short fiber reinforcement (SFR), continuous fiber reinforcement (CFR), powder addition reinforcement (PAR), vibration-assisted FFF (VA-FFF) methods, as well as annealing. In this paper, the details of all these reinforcement techniques are reviewed. The abilities of each method in improving tensile, flexural, and compressive strength are discussed.

## 1. Introduction

Currently, additive manufacturing (AM), also often called rapid prototyping (RP) and three-dimensional (3D) printing, has gained popularity owing to its flexibility in the fabrication of 3D parts with complex geometries and customized designs [1,2]. In this technique, a 3D part can be produced by building up multiple layers of material until the desired form is achieved [3]. So far, several AM techniques have been developed and used worldwide, such as stereolithography (SLA) [4], selective laser sintering (SLS) [5], laminated object manufacturing (LOM) [6], and fused filament fabrication (FFF) [7]. Among these techniques, FFF, interchangeably called fused deposition modeling (FDM), has been recognized as one of the most practical and promising, owing to its reliability and affordability [8,9].

**Figure 1:**
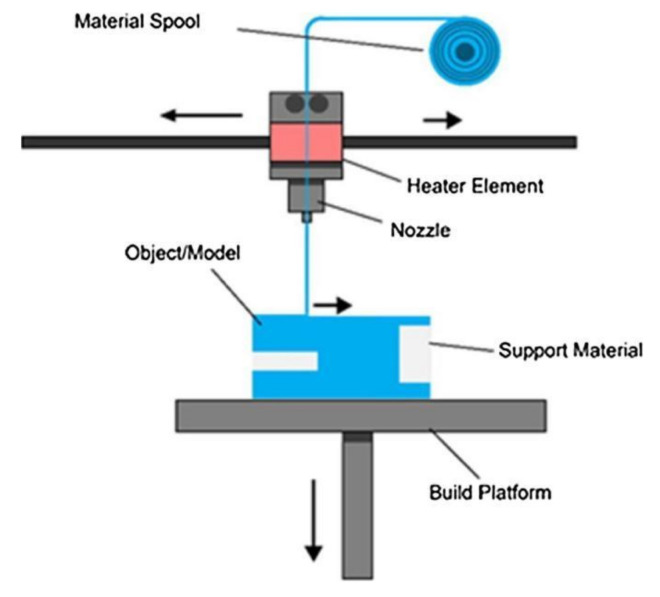
The principle of fused filament fabrication process. Reprinted with permission from [12]. Copyright 2018, Elsevier.

In the FFF process, a 3D part can be constructed by building up multiple layers of material deposited according to a sliced 3D model generated by computer-aided design (CAD) software [10]. As shown in Figure 1, FFF utilizes a spooled polymeric filament as the raw material, heating it up to its semi-molten state and then depositing it layer-by-layer through the extrusion nozzle of the printer to produce a 3D part. For this purpose, the FFF-based 3D printer is equipped with a movable printing bed or build platform that can travel down a few micrometers to allow the deposition of the subsequent layer of material onto the previously solidified layer, until the printing process is accomplished [11].

FFF has been widely used for various applications, from being the supporting unit for rapid prototyping processes to being used for fabrication of custom-designed functional products [13]. As noted earlier, for example, the work of Guo and Leu successfully manufactured an intake manifold of a 600 cc formula automotive engine made from carbon fiber composite material, processed using FFF [14]. Meanwhile, FFF has also been used for the fabrication of ankle foot orthoses from ABS M30 and polypropylene (PP), as reported in the study of Banga et al. [15,16].

Owing to the technological developments and achievements gained so far, FFF has become a competitor to conventional manufacturing techniques [17]. However, there are several limitations concerning FFF-processed products. For example, it has long been recognized that FFF-processed parts are generally weaker than those produced by casting and injection molding [18,19,20]. According to a previous study, the tensile and compressive strengths of 3D-printed ABS (acrylonitrile butadiene styrene) was only 73% and 80–90% of those of injection-molded ABS, respectively [21]. Meanwhile, the tensile strength of FFF-processed PLA (polylactic acid) was about 70–90% of casted PLA [22,23]. All these findings suggest that anisotropic properties and weak inter-raster bond strength were among the causes of such a low mechanical reliability of FFF-processed materials [21,24,25], as well as the cavities that were formed inside the interior of the 3D-printed parts [18,21,26,27,28].

Over the last decade, researchers have conducted many studies to improve the mechanical properties of FFF-processed polymeric materials. Several reinforcement techniques have been developed—for instance, the optimization of printing parameters, the addition of fibrous and powdered material into the printed polymer, and the application of vibrations during the printing process, as well as post-processing treatments such as annealing. However, the studies concerning all these methods have not yet been collated, leading to difficulties for readers in choosing the appropriate techniques for certain applications.

In this article, an overview of the reinforcement techniques that have so far been applied in FFF-processed polymeric materials is presented. Firstly, the principles of each method are briefly presented. After this, the benefits and limitations of the methods are then tabulated and discussed. With this article, it is expected that readers will be able to gain insight into recently developed techniques for reinforcing and improving the mechanical properties of FFF-processed polymeric materials.

## 2. Adjustments of the Printing Parameters Used in FFF

Adjusting the parameters used in FFF printing might be the simplest way to improve the mechanical properties of FFF-processed parts. The tensile strength (UTS), compressive strength (CS), and bending or flexural strength (FS) of FFF-processed part can be tailored by optimizing several of the parameters used in the printing process, such as the build orientation, layer height, raster width, raster angle, infill percentage, air gap, and extrusion temperatures.

In FFF processing, the build orientation is defined as the nozzle direction applied in the 3D printer when it is operated to deposit layers of material to build up the printed parts [29,30]. There are three types of build orientation in FFF process, i.e.,

- Horizontal or *xyz* build, where the nozzle moves along the *x*-axis that represents the length, the *y*-axis that represents the width, and the *z*-axis that represents the thickness of the printed parts;
- Vertical or *xzy* build, where the nozzle moves along the *x*-axis that represents the length, the *z*-axis that represents the width, and the *y*-axis that represents the thickness of the printed parts;
- Perpendicular or *zxy* build, where the nozzle moves along the *z*-axis that represents the length, the *x*-axis that represents the width, and the *y*-axis that represents the thickness of the printed parts.

**Figure 2:**
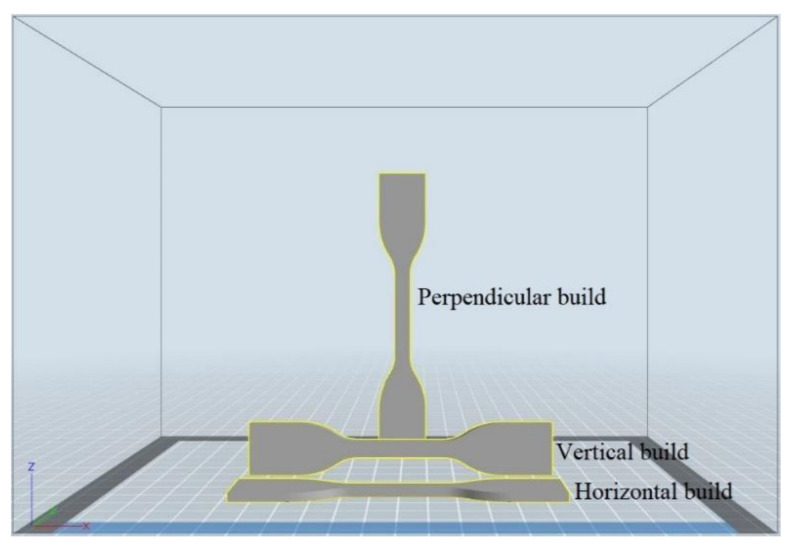
Graphical representation of various build orientations.

Figure 2 shows a graphical representation of tensile specimens printed with horizontal, vertical, and perpendicular builds.

**Figure 3:**
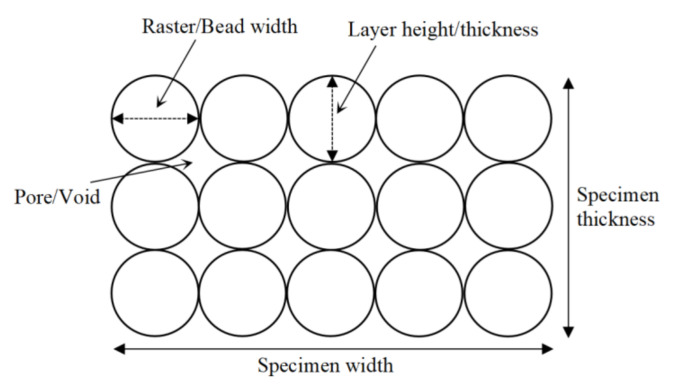
Schematic illustration of the layer height and raster width.

The layer height and the raster width are also considered the other critical parameters that can determine the mechanical properties of FFF-processed parts. In principle, the layer height and raster width correspond to the thickness of the deposited material printed along the *x* and *y*-axis during the printing process [29,31], as illustrated in Figure 3. In practice, this parameter is determined by the nozzle diameter of the FFF-based 3D printer [29].

**Figure 4:**
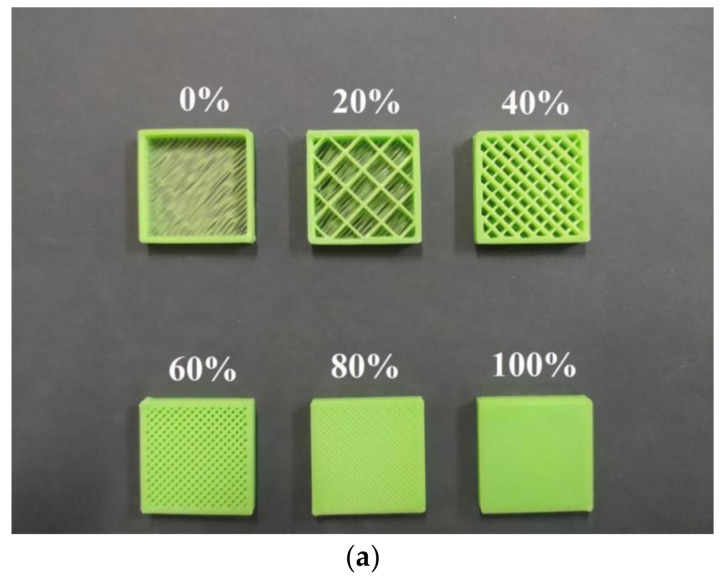

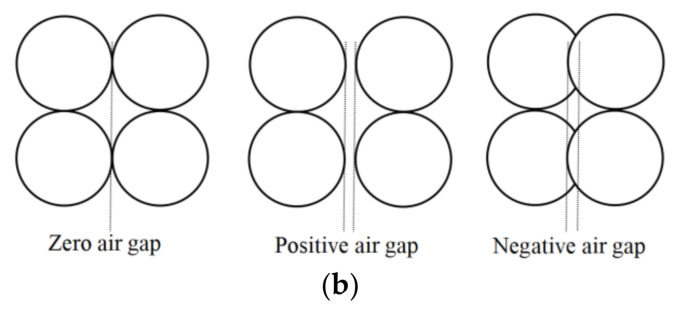
(**a**) infill percentage, and (**b**) schematic illustrations of an air gap in FFF-processed material.

Prior to FFF processing, it is also important for the operator to set up the infill percentage applied during the printing process [32]. Figure 4 illustrates the relationship between this parameter and a so-called air gap on the deposited material of the printed part. As indicated in Figure 4b, this air gap can be defined as the space between the two adjacent rasters, and this parameter is obviously determined by the infill percentage applied during the printing process [33,34]. As seen in Figure 4a, the infill percentage can be adjusted from 0 to 100%. Once a low infill percentage was applied, there would be a separating space between the two adjacent rasters in the printed part [35]. Such a space could be narrowed, thereby reducing the inter-raster gap, by increasing the infill percentage up to its maximum, i.e., 100%. Printing with low infill percentages would lead to the formation of printed parts with a positive air gap, as indicated in Figure 4b, where the gap can be clearly seen between the two adjacent printed rasters. With a certain value of infill percentage (100%), a zero-air gap can be formed, in which the surfaces of the two adjacent rasters touch one another. A negative air gap could theoretically be achieved by printing the part with a >100% infill percentage. To achieve an infill density beyond 100%, several slicing strategies can be taken, such as:

- increasing the raster width;
- increasing the infill overlap percentage and the perimeter overlap percentage.
- using the filling gaps option in the space between the walls.

All the strategies mentioned above can be easily executed using several slicing software, such as Cura and Simplify.

**Figure 5:**
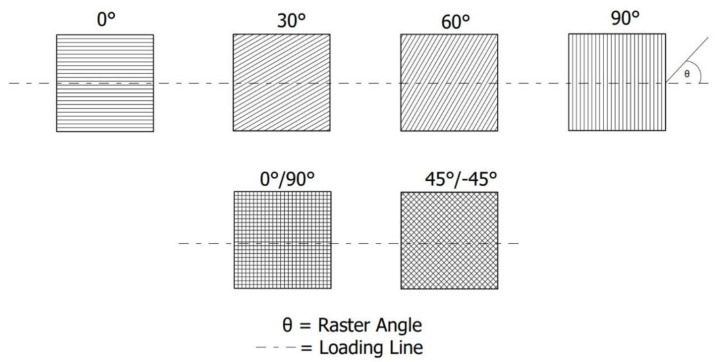
Schematic illustration of various raster angles.

Finally, the raster angle has long been considered as one of the most important parameters in FFF processing. This angle is formed by the linear direction traveled by the nozzle during the deposition of the molten filament relative to the axis of the load applied to the printed material [29,31,36]. As illustrated in Figure 5, the raster angles can be varied from 0 to 90°. In some cases, a configuration with a combination of raster angles, occasionally called criss-cross raster angles, has been applied and studied in FFF-processed materials. Table 1 summarizes recent studies concerning the influence of printing parameters on the mechanical properties of FFF-processed polymeric materials.

As shown in Table 1, it is obvious that the raster angle, build orientation and air gap have significant impacts on the ultimate tensile strength (UTS) of FFF-printed ABS [21,37,43,45,46]. Sood et al. also reported that the layer thickness and the raster width also determined the UTS values of FFF-processed ABS [29]. In addition, Álvarez et al. stated that the infill percentage and extrusion temperature affected the strength of FFF-processed ABS [45]. Furthermore, the works of Dawoud et al. and Cantrell et al. demonstrated that the combination of criss-cross raster angle and negative air gap could yield a printed ABS with a higher UTS than that with the unidirectional raster angle [10,47]. On the other hand, the research conducted earlier confirmed the significant roles of the raster angle, raster width, layer thickness, and build orientation on the strength of FFF-processed PLA [31,43].

As summarized in Table 1, the compressive strength (CS) of FFF-processed materials is also determined by the build orientation [21,39], as well as the raster angle, raster width and air gap applied in the printing of the material [40]. Notably, to achieve a 3D-printed ABS with the highest CS value, a horizontal build should be applied during the printing process, instead of a vertical one [21,39].

The works of Es-Said et al. and Durgun and Ertan pointed out the importance of raster angle and build orientation in determining the flexural strength (FS) of FFF-processed ABS [36,42]. As reported earlier, the application of criss-cross raster angles of 0°/90° and a negative air gap resulted in a printed ABS with the highest flexural strength [10]. In the case of FFF-processed PLA, a study conducted by Chacón et al. also showed the importance of build orientation and printing speed on the flexural strength of a printed PLA [48]. Finally, the extrusion temperature should also be selected appropriately as it also determines the flexural strength of the printed PLA; as highlighted by Kuznetsov et al., the flexural strength increases as the extruder temperature increases, until reaching a maximum strength at 250 °C [49].

Based on all these findings, it can be concluded that the build orientation, raster angle, and layer thickness are among the most important or critical parameters that influence the mechanical properties of FFF-processed polymeric materials. The infill percentage and air gap are usually considered the standard parameters in FFF, and therefore are often called fixed parameters. Meanwhile, the extruder temperature and printing speed are among the operation settings that are dependent on the type of filament material used in the FFF process.

## 3. Short Fiber Reinforcement (SFR) Method

**Figure 6:**
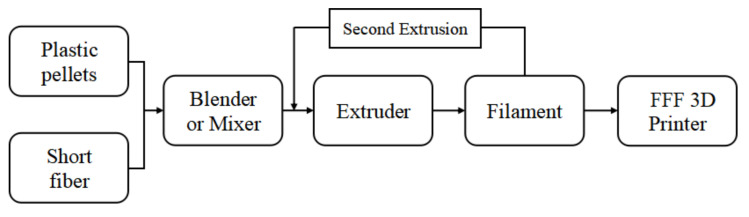
The flow-chart of the SFR process.

In this method, short fibers are incorporated into the polymeric filament used for FFF and act as fillers that are capable of strengthening the material printed from this composite filament [50]. As shown schematically in Figure 6, the composite filament used in this method is first prepared by blending the polymeric pellet material together with short fibers whose length is in the range of ~0.1–3 mm prior to extrusion. A refining process can be conducted by re-blending the filament, so that a filament with a higher bulk density can be achieved [28]. Up to recently, several types of fibers have been used as the reinforcement filler in the SFR method, for instance, carbon fiber [18,28], jute fiber [51], glass fiber [52,53,54], and graphene [20]. Table 2 shows the recent progress in research applying the SFR method in FFF processing.

In general, the mechanical properties of printed parts increase with the application of the SFR method. In their work, Tekinalp et al. reported an increase in ultimate tensile strength (UTS) of 85% in FFF-processed ABS reinforced by short carbon fiber [18]. Similarly, the work of Ning et al. on printed ABS demonstrated an increase of its UTS value by 20% and its elastic modulus by 30% with the addition of 5% and 7.5% carbon fiber, respectively [28]. Meanwhile, the use of 5 to 40 wt.% glass fiber as a filler could also improve the tensile strength of FFF-processed ABS/PA6 composites by 117% [52]. However, material embrittlement could also occur as a result of glass fiber addition, as indicated by the decreased value of the material elongation-at-break from 220 to 10%. In the case of FFF-processed PLA, however, the addition of 15 wt% of glass fiber could only increase the UTS by 2.2%, indicating that the PLA did not bind well to the glass fibers [54]. Meanwhile, the addition of 30 wt% of glass fiber into polypropylene (PP) was able to enhance the tensile strength and modulus of this material by 40% and 30%, respectively [53].

Despite these promising results, several studies have shown that the use of the SFR method could deteriorate the mechanical properties of FFF-processed materials. Despite increasing the fracture strength by 28%, the addition of jute fiber decreased the UTS value of FFF-processed ABS by 9% prepared with a horizontal build [51]. Additionally, the work of Dul et al. reported a decrease in the UTS value with increases in the xGnP (graphene) fiber content of ABS. In this case, the lowest UTS was achieved when the ABS was printed with 8 wt% of xGnP [20].

Considering all the findings mentioned above, it can be summarized that short fiber reinforcement could be a promising method for improving the mechanical properties of printed parts, as shown from the remarkable improvement in UTS values [18,28,52,53]. However, the selection of the base material and type of reinforcing fiber must be considered carefully, as shown by some results which indicated a strength deterioration after the application of certain types of reinforcing fiber [20,51]. Additonally, with an increase in fiber content, nozzle clogging is likely to occur, as mentioned in the work of Tekinalp et al. [18].

## 4. Continuous Fiber Reinforcement (CFR) Method

**Figure 7:**
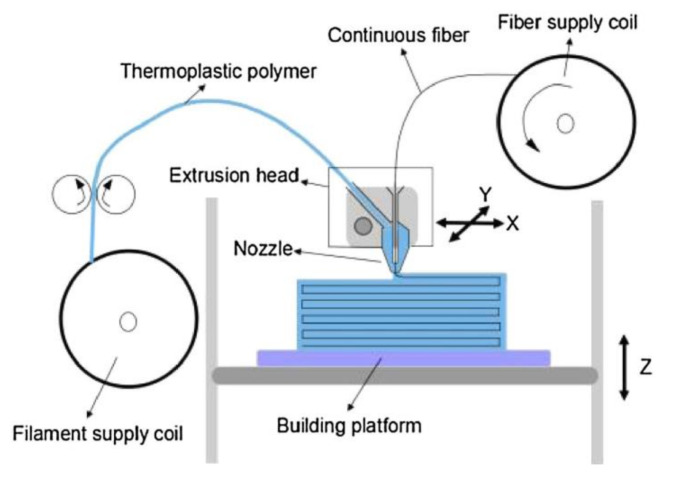
FFF process with the CFR method. Reprinted with permission from [60]. Copyright 2016, Elsevier.

The continuous fiber reinforcement (CFR) method is carried out by combining two types of materials, i.e., the polymeric filament and fiber, during the FFF process to improve the mechanical properties of the printed parts. Unlike the SFR method, the fiber in the CFR technique is added to the polymer matrix during the printing process [55,56]. As shown in Figure 7, the CFR method is equipped with a two-inlet extrusion head which allows the blending and extrusion of the thermoplastic polymeric filament together with the reinforcement fiber during the FFF process. For this purpose, a conical extrusion nozzle is often used to improve the uniformity of the polymeric matrix and fiber blending during the printing process [57]. Up to now, there have been several types of fibers utilized in the CFR process, e.g., carbon fibers [57,58,59,60], flax fibers [61], glass and Kevlar fibers [62], and jute fibers [63]. Table 3 summarizes the recent progress of research conducted on FFF processing with the CFR method.

In general, the use of the CFR method could improve the mechanical properties of FFF-processed polymeric materials. According to the work of Matsuzaki et al., the use of carbon fiber as the filler could increase the UTS and elastic modulus of FFF-processed PLA by 435% and 599%, respectively [63]. Meanwhile, the work of Li et al. showed that the addition of the previously modified fiber using methylene dichloride solution could increase the UTS and flexural strength of printed PLA specimens by 225% and 194%, respectively [57]. Similar to the work of Li et al., the research carried out by Tian et al. demonstrated that the flexural strength and elastic modulus of the PLA with CFR increased once higher extrusion temperatures were used [60]. In this case, FFF-processing at a temperature of 240 °C yielded CFR-PLA with a flexural strength that was about 70.3% higher than that obtained at an around-melting point temperature, as obtained by Li et al. Furthermore, the carbon fiber content has been recognized as an important factor determining the mechanical properties of reinforced materials. Li et al. noted that the UTS of PLA with CFR increases with increasing fiber content [64]. In their work, it was also noted that a maximum UTS of 106.3 MPa could be achieved when the PLA contained 15 wt% fiber.

The work of Heidari-Rarani et al. reported the use of additional chemical treatments alongside CFR-PLA filament preparation [59]. Interestingly, such treatments could increase the UTS and elastic modulus values of the printed CFR-PLA by 36% and 208%, respectively, and decrease the failure strain by 62% compared to pure PLA. This treatment also increases the flexural strength and modulus by 109% and 367%, respectively. The work of Le Duigou et al. utilized a flax fiber as the filler, with the filler having a diameter of 482 ± 30 μm, to reinforce the PLA filament [61]. When printed at a 0° raster angle and 30 vol% fibers, the UTS and stiffness of the printed PLA increased by 4.5 and 7 times, respectively. On the other hand, Mangat et al. reported that the use of silk and sheep wool as the filler could not improve the mechanical properties of the printed CFR-PLA, as indicated from its flexural strength, which could only reach 24.58 MPa or 52% lower than that of non-reinforced PLA [65]. The use of CFR to improve the mechanical properties of FFF-processed nylon has been studied by Naranjo-Lozada et al. In their work, it was shown that the maximum UTS could be achieved in CFR nylon with a 54% carbon fiber content [66]. Meanwhile, a study conducted by Dickson et al. ranked carbon fiber as the most suitable filler, followed by glass and Kevlar fibers, for producing high-strength FFF-processed CFR nylon [62]. In addition, it was also shown that the raster pattern can affect the strength of printed materials as well. In this case, the printed material with an isotropic pattern had a greater tensile strength and elastic modulus than that printed with a concentric pattern. The results from this study showed that the maximum UTS was obtained using carbon fiber, at 254% higher than that of pure nylon. Nevertheless, the failure mode was a brittle fracture. In addition to this, the maximum FS was also obtained using carbon fiber, at 496% higher than that of pure nylon. This work has shown that both tensile and flexural strength increase as fiber volume increases.

**Figure 8:**
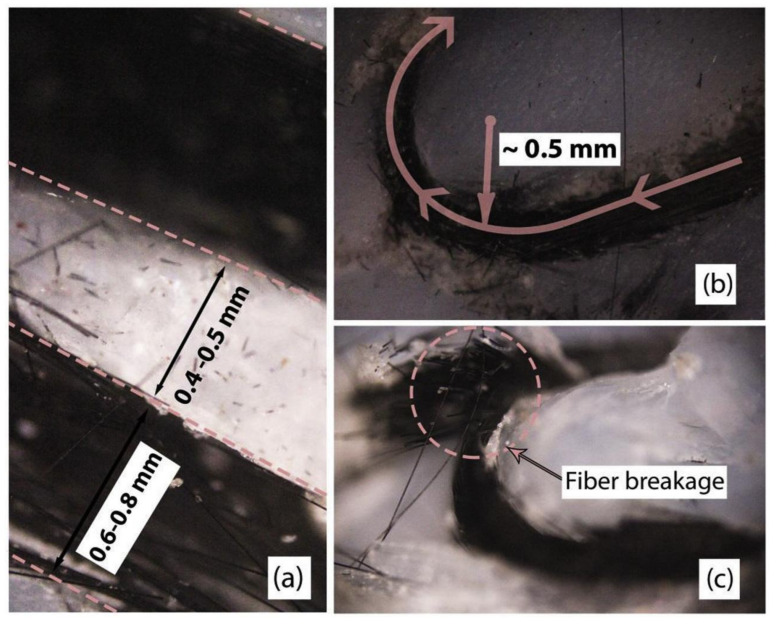
(**a**) Minimum gap in CFR, (**b**) return radius, and (**c**) fiber breakage during returning. Reprinted with permission from [59]. Copyright 2019, Elsevier.

From all these previous works, it can be summarized that the CFR method has a better performance in terms of resultant mechanical properties than the SFR method. However, this method has a major disadvantage: fiber breakage tends to occur during the printing process (Figure 8c). This may happen primarily due to the curve path of the nozzle when it reaches the edge of the geometry and turns around to form a U path, known as the return radius (Figure 8b). According to Heidari-Rarani et al., a minimum gap of 0.4–0.5 mm should be applied between the two parallel paths of the fibers during the printing process [59], as illustrated in Figure 8a. Due to this phenomenon, a negative air gap cannot be applied in the CFR method.

## 5. Powder Addition Reinforcement (PAR) Method

Similar to the SFR technique explained in Section 3, the principle of the powder addition reinforcement (PAR) method relies on the strengthening mechanism of the powder particles that are added to the polymer matrix material prior to the FFF printing process. The powder particles are added to the matrix material through the extrusion process (see Figure 6) to produce a composite filament material consisting of two types of materials. Table 4 summarizes the results of recent studies concerning FFF-processed materials that have been prepared utilizing the PAR method. Several powders have been used so far—for instance, metal powder [51,52,67,68,69,70], montmorillonite (OMMT) [71], rice straw (RS) powder [72], wood powder [73], and multi-wall carbon nanotubes (MWCNT) [74].

In general, the PAR method can enhance the mechanical properties of FFF-processed materials. The work of Karsli et al. demonstrated an increase in the tensile strength of the ABS/PA6 printed blend of 15% with the addition of CaCo_3_ powder up to 5 wt% [52]. However, it is also noted that the elongation of such composite materials decreased from 220 to 62%, proving that the printed material became brittle with the powder addition. Several earlier works have also shown the benefits of using several types of powders to improve the mechanical properties of printed ABS, such as TiO_2_ [51], montmorillonite (OMMT) [71], Al and ZrB_2_ [67], and multi-wall carbon nanotubes (MWCNT) [74] powders. However, it is also recognized that this powder addition could potentially lead to embrittlement of the printed ABS, which in the end decreases the elongation of the printed material [51]. In addition to its tensile properties, the addition of OMMT, TiO_2_, and ZrB_2_ powders could also be seen to improve the flexural strength of the printed ABS [51,68,72].

As reported in several studies, however, the addition of several types of powders was not able to improve the mechanical properties of FFF-processed materials. The works of Masood and Song and Nikzad et al. noted decreased tensile strength, elastic modulus, and elongation in the printed nylon and ABS [68,69]. The addition of wood powder also decreased the tensile strength of the printed PLA, although it increased the water absorption of this material [73]. Despite being able to reduce bacterial growth, the addition of AgSMW powder also decreased tensile strength and strain-at-break of the printed PLA [70]. Meanwhile, the addition of a low content of rice straw powder decreased the flexural strength of the printed ABS [72]. As also noted in this study, the flexural strength reached the same value as the printed ABS once supplemented with 15 wt.% rice straw powder.

**Figure 9:**
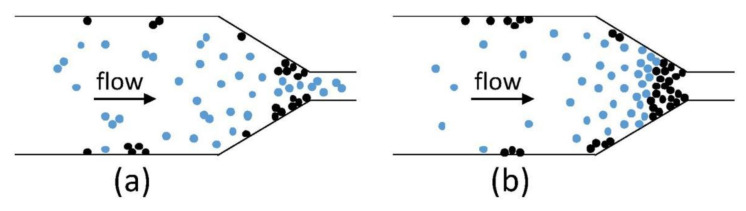
A schematic illustration of nozzle clogging in the PAR method, (**a**) reduced flow due to curvature, (**b**) agglomeration of powder; the blue and black dots in the figure correspond to the polymer matrix and the metal particles, respectively. Reprinted with permission from [75]. Copyright 2018, Elsevier.

Based on the works presented above, the PAR method could be a promising method for improving the mechanical properties of FFF-processed parts. However, the selection of an appropriate base material and reinforcing powder must be considered carefully, as many published works also showed decreased strength of FFF-processed material with the addition of certain powder types [68,69,70,72,73]. Similar to SFR, nozzle clogging has also been recognized as a serious problem that might be encountered in the PAR method due to the curvy shape of the nozzle, which might cause flow obstruction and agglomeration of powder particles at the nozzle tip [75], such as that illustrated in Figure 9.

**Figure 10:**
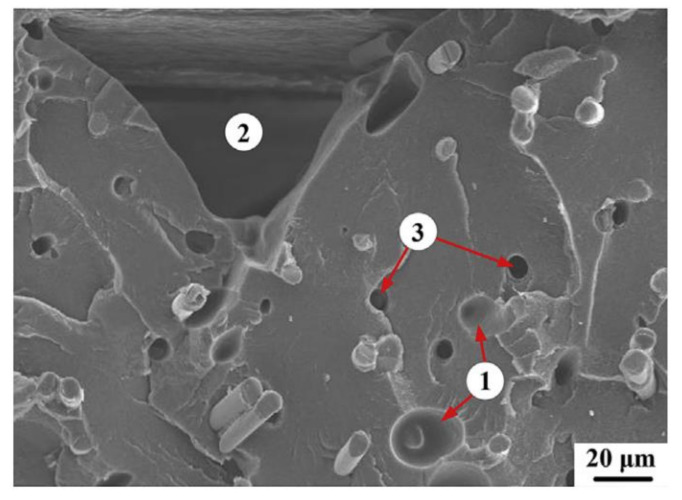
Illustration of different categories of porosity in a short fiber reinforced specimen. Reprinted with permission from [28]. Copyright 2015, Elsevier.

Besides nozzle clogging, the distribution of the fraction and the sizes of the powder attached to the polymeric matrix could also determine the tensile strength, modulus, and elongation of FFF-processed composite materials [68]. In addition, the presence of gaps or voids around the filler particle has been recognized as a weak point of printed materials [72], which in the end lead to a decrease in the mechanical properties of the printed materials. As shown in Figure 10, the work of Ning et al. [28] revealed three types of voids that could be formed in FFF-processed composite materials using the PAR or SFR methods, i.e.,

1. gas-evoluted pores that form during the extrusion process of the blend filament;
2. physical gaps that form at the inter-raster region of the printed materials;
3. holes that form around the pulled-out fibers, existing on the fracture interface of the printed parts.

## 6. Vibration-Assisted FFF (VA-FFF) Method

The presence of voids or pores at the interior of printed parts has been recognized as one of the major limitations of the FFF technique. With the presence of these pores, the load-bearing area of the printed part is reduced and, consequently, the mechanical properties of such FFF-processed materials are compromised. Several studies have therefore been performed in an attempt to minimize porosity, as well as the sizes of pores that are formed in FFF-processed materials.

**Figure 11:**
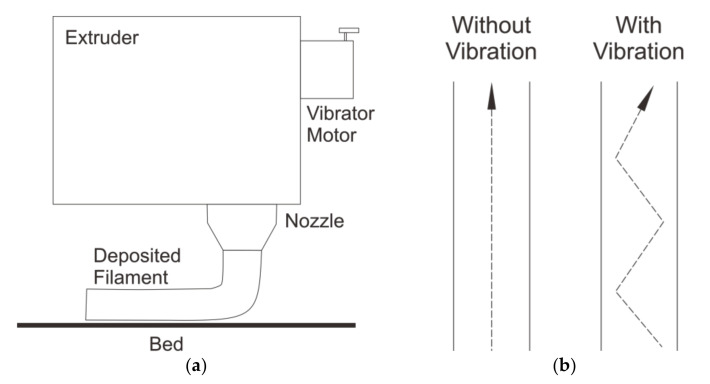
(**a**) A schematic illustration of the application of a vibrator at the extrusion head of an FFF 3D printer, (**b**) material deposition path with or without the application of vibration.

Recently, vibration-assisted FFF (VA-FFF) has been introduced as a method for reducing the porosity of FFF-processed parts. As illustrated in Figure 11a, this method utilized a vibrator-equipped nozzle or extrusion head in an FFF 3D printer. When the printer was in use, the extruder would shake and allow a more uniform deposition of material into the space at the inter-raster region of the printed material. As also indicated in Figure 11, the use of a vibrator could alter the deposition path from a straight to a zigzag pattern (Figure 11b). Table 5 summarizes the findings obtained from research concerning the mechanical properties of printed materials processed using the VA-FFF technique.

The works of Keles et al. and Jiang et al. demonstrated VA-FFF as a promising technique for improving the mechanical properties of FFF-processed polymers. In their work, Keles et al. showed a reduction in the total porosity of short carbon fiber-reinforced ABS (SCFR-ABS) parts from 13 to 10%, and the improvement of its fracture strength, tensile strength, and strain-at-break by >10% in this material by applying a vibrating nozzle during the printing process. It is also reported from this study that the use of a vibrating nozzle could also slightly increase the elastic modulus of the printed material from 2.5 ± 0.1 GPa to 2.7 ± 0.1 GPa [76]. Similarly, Jiang et al. reported an increased tensile strength of PLA of 50% once this material was printed along a z orientation using a vibrating nozzle [77].

**Figure 12:**
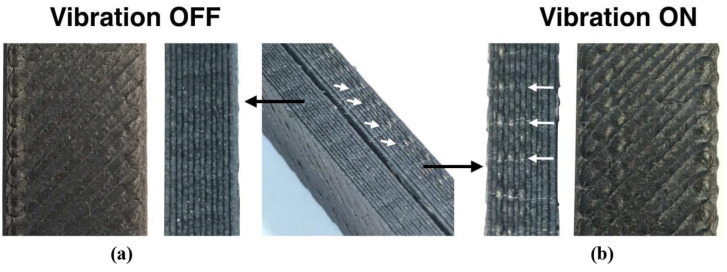
The surface of FFF-processed specimens: (**a**) without and (**b**) with vibrations. The white arrows in the figure show the crests of the vibration waves, resulting in waviness beyond the contour position. Reprinted with permission from [76]. Copyright 2018, Emerald Publishing Limited.

Despite promising results, VA-FFF has not yet been used widely for many applications. Moreover, the influence of some operational parameters on the properties of the printed material prepared using this technique has not so far been well defined. The limitations of VA-FFF have also been recognized—for instance, the distortion of part dimensions and the rougher or wavy surfaces of printed parts [76], such as those shown in Figure 12. Therefore, more intensive future studies will be necessary to further explore and develop VA-FFF processing for the manufacturing of polymeric parts.

## 7. Annealing Post-Processing

Non-uniform cooling rates over the deposited layers of printed materials have also been considered another shortcoming of the FFF technique [78,79]; this phenomenon could lead to the formation of premature cracks and, subsequently, failure due to thermal-induced residual stress of the FFF-processed material [80]. To prevent this problem, annealing has so far been the preferred method following the printing process to achieve an even distribution of heating and a uniform cooling rate over the whole body of the printed part. With this approach, the bonding quality of the deposited raster can be improved, the porosity of the printed part can be reduced and, ultimately, the mechanical properties of the FFF-processed part can be enhanced [81].

**Figure 13:**
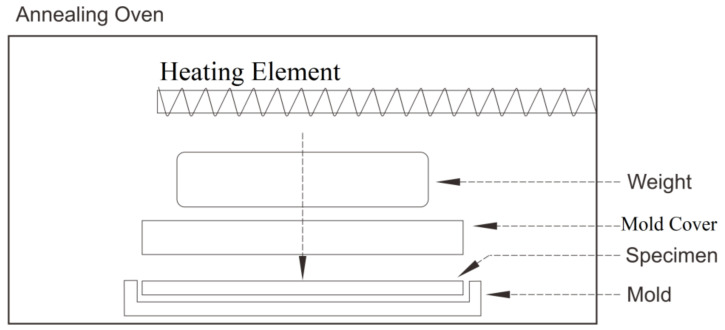
The experimental setup for pressure-assisted annealing of an FFF-processed polymeric part.

In a recent work, annealing of FFF-processed part was performed using a forced convection oven, as reported in the work of Jo et al. [81]. As shown in Figure 13, the printed part was inserted into place between the mold and the mold cover plate. A weight could also be added on the top of the mold cover to increase local pressure over the specimen body as well as the inter-raster area inside the material. During the annealing process, both the molds and the specimen were then heated up to a specific temperature in the oven.

**Figure 14:**
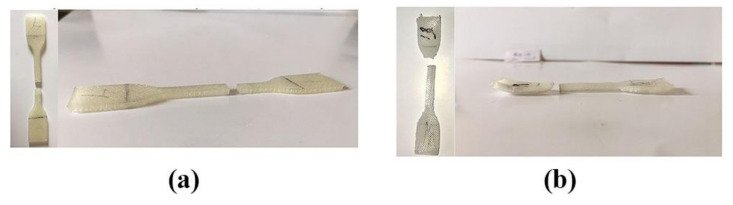
The tensile specimen (**a**) before, and (**b**) after the annealing process. Reprinted with permission from [82]. Copyright 2020, Emerald Publishing Limited.

Table 6 summarizes recent works on the annealing of FFF-processed polymers. The work of Jo et al. demonstrated that the tensile strength of FFF-processed PLA increased by 5% once annealed at 160 °C for 30 s. By prolonging the duration of annealing up to 120 s and applying a pressure of 19 N, the tensile strength of such printed materials could be increased by up to 10.2% [81]. In contrast, Behzadnasab et al. reported that annealing temperatures from 60 to 120 °C for 30 to 120 min decreased the material’s strength and had no significant impact on the elastic modulus of FFF-processed PLA—possibly due to molecular degradation in this material [82]. It is also important to note that the annealing caused the specimen to slightly bend, such as is shown in Figure 14, due to the recrystallization process caused by the reheating and cooling process, which could yield residual stresses over the printed parts. Basically, annealing could be considered a promising technique for improving the mechanical properties of FFF-processed polymers, owing to its simplicity and low-cost of operation; however, the two recently published articles summarized above demonstrate conflicting results regarding the use of this treatment for FFF-processed PLA. Further studies are therefore required to gather a deeper understanding of this reinforcement mechanism in printed polymers using annealing.

## 8. Concluding Remarks

In this article, an overview of several techniques that can be used for improving the mechanical properties of FFF-processed polymeric materials is presented. In summary, the adjustment of FFF-processing parameters has so far been used as the simplest way to enhance the tensile properties of printed materials. Therefore, it is posited in this review article that these printing parameters should be adjusted appropriately to achieve an FFF-processed part with the desired mechanical properties. Meanwhile, several reinforcement methods have been recognized that can be performed complementarily to FFF processing, such as short fiber reinforcement (SFR), continuous fiber reinforcement (CFR), powder addition reinforcement (PAR), vibration-assisted FFF (VA-FFF), and annealing. The advantages and disadvantages of each these reinforcement methods are presented in Table 7.

According to the results of the literature study presented in this article, the capability of the different reinforcement methods for improving the strength of FFF-processed materials could be ranked from highest to lowest as follows: CFR, SFR, annealing, PAR, and VA-FFF. The CFR method can be used to produce a 3D-printed part with the highest possible strength owing to a large amount of filler that can act as the skeleton of the printed material. With this technique, it is also possible to add the filler at a volume percentage greater than that of the polymer matrix.

In the case of the SFR and PAR methods, the filler content should not be more than ~40% as otherwise it may lead to nozzle clogging. Unlike VA-FFF, both the SFR and PAR methods cannot be used to reduce porosity as the filler could occupy the spaces between the layers of the printed material. Although SFR and PAR have been used since almost 20 years ago, the studies concerning the use of these techniques have so far been focused on the combination of polymer matrix and filler particles and on variations in the polymer matrix and filler fractions. Future work in this field can be directed towards the optimization and development of filler addition during the printing of polymeric materials using FFF. With this approach, the use of multi-filler is employed to achieve the production of an FFF-processed polymer with appropriate mechanical properties. Meanwhile, it is also noted that long-term use of the VA-FFF method may reduce the accuracy of and even damage the printer. Therefore, further research is needed to support the effectiveness of this method. Similar to VA-FFF, the effectiveness of annealing as a post-treatment of FFF is also still unclear due to the lack of research performed using this method.

## Figures and Tables

**Table 1:** Research on printing parameters.

Authors	Materials		Methods	Dimensions & Testing Standards			Outcomes
	Base	Addition		Tensile	Flexural	Compressive	
Es-Said et al. (2000) [36]	ABS	-	Variation of raster angle	ASTM D638	ASTM D790	-	UTS: ~20.6 MPa, at 0° raster angle. FS: ~44.4 MPa, at 0° raster angle.
Rodriguez et al. (2001) [37]	ABS	-	Variation of the air gap	ASTM D3039	-	-	The printed parts had a lower modulus and strength by 11–37% and 2–57%, respectively, compared to the single filament.
Ahn et al. (2002) [21]	ABS	-	Variation of raster angles for tensile test; Horizontal & vertical builds for compression, compared to the samples obtained from injection molding (IM)	ASTM D3039	-	Not standardized	UTS IM: 26 MPa, UTS FFF: 10–3% of IM; The greatest UTS occurred at the sample with a 0° angle. CS IM: ~40 MPa, CS FFF: 80–90% of IM. The greatest CS occurred at the sample with the horizontal build.
Bellini and Güçeri (2003) [38]	ABS	-	Variation of build orientations and criss-cross raster angle	ASTM D5937	-	-	When the tensile load was in-line with the fiber orientation, the tensile strengths of each sample was not much different from one another. If the fiber orientation was not in-line to the tensile load applied, the load bearing role was carried out by the interlayer bonding of adjacent filaments.
Lee et al. (2007) [39]	ABS	-	Vertical & horizontal build	-	-	ASTM D695	CS: 41.26 MPa at a horizontal build—11.6% higher than the CS of the sample prepared with a vertical build.
Sood et al. (2010) [29]	ABS	-	Variation of layer thickness, orientation, raster angle, raster width, and air gap	ISO R527	ISO R178	-	UTS: ~39.24 MPa, at 0.127 mm layer thickness, 30° orientation, 60° raster angle, 0.4064 mm raster width, and 0.0080 mm air gap. FS: ~18.09 MPa, at 0.127 mm layer thickness, 30° orientation, 60° raster angle, 0.4064 mm raster width, and 0.0080 mm air gap
Sood et al. (2012) [40]	ABS	-	Variation of layer thickness, orientation, raster angle, raster width, and air gap	-	-	ISO R291	CS 17.475 MPa, at 0.254 mm layer thickness, 0.036° orientation, 59.44° raster angle, 0.422 mm raster width, and 0.00026 mm air gap.
Jami et al. (2013) [41]	ABS	-	Variation of build orientation on dynamic compressive strength	-	-	Not standardized	Dynamic CS: ~80 MPa, at vertical orientation.
Durgun and Ertan (2014) [42]	ABS	-	Variation of build orientation and raster angle (H = Horizontal, V = Vertical, P = Perpendicular)	ISO R527	ISO R178	-	UTS: ~35 MPa, at H-0°, but lower modulus (~2 GPa), Elastic Modulus: ~2.5 GPa, at P-90°, but lower strength (~20 MPa). FS: ~60 MPa, at V-0°.
Lanzotti et al. (2015) [43]	PLA	-	Variation of build orientation, raster angle, and layer thickness	ASTM D638	-	-	UTS: 53.59 MPa at 0° raster angle with 0.15 mm layer thickness and horizontal build.
Ziemian et al. (2015) [44]	ABS	>-	Variation of raster angle, compared to injection molding (IM)	ASTM D638	-	-	UTS IM: 27 MPa UTS FFF: 25.15 MPa, at 0° angle.
Álvarez et al. (2016) [45]	ABS	-	Variation of infill percentage, build orientation, and extrusion temperature	ASTM D638	-	-	UTS: 34.57 MPa, at 100% infill, horizontal build, 0.2 mm layer thickness, and 250 °C extrusion orientation, and extrusion temperature.
Dawoud et al. (2016) [10]	ABS	-	Variation of criss-cross raster angle and air gap, compared to IM	ISO R527	ISO R178	-	UTS IM: 37.7 MPa. UTS FFF: 34.3 MPa, at 45°/−45° raster angle with an air gap of −0.05 mm. FS IM: 72.5 MPa. FS FFF: 64 MPa, at 0°/90° raster angle with an air gap of −0.005 mm.
Rankouhi et al. (2016) [46]	ABS	-	Variation of layer thickness, raster angle, and number of layers	ASTM D638	-	-	UTS: 39.4 MPa, at 0° raster angle, 0.2 mm layer thickness, total 35 layers. The UTS increase as the number of layer increase.
Cantrell et al. (2017) [47]	ABS & PC	-	Variation of criss-cross raster angle and build orientation	ASTM D638	-	-	Yield Strength (ABS): 33.5 ± 0.5 MPa, at 0°/90° horizontal build. Yield Strength (PC): 61.1 ± 0.5 MPa, at 45°/−45° vertical build.
Chacón et al. (2017) [48]	PLA	-	Variation of build orientation, layer thickness, and printing speed	ASTM D638	ASTM D790	-	UTS: ~88 MPa at horizontal build, 0.06 mm layer thickness, and 80 mm/s printing speed. FS: ~62 MPa at vertical build, 0.06 mm layer thickness, and 80 mm/s printing speed.
Rajpurohit and Dave (2018) [31]	PLA	-	Variation of raster angle, layer thickness, and raster width	ASTM D638	-	-	UTS: 47.3 ± 2.69 MPa at 0° raster angle, 0.1 mm layer height, and 0.6 mm raster width.
Kuznetsov et al. (2020) [49]	PLA	-	Variation of extrusion temperature and feed rate	-	Not standardized	-	FS: 71.1 MPa, at 250 °C extrusion temperature, 25 mm/s printing speed, and without cooling from a fan.

**Table 2:** Research on Short Fiber Reinforcement.

Authors	Materials		Methods	Dimensions & Testing Standards			Outcomes
	Base	Addition		Tensile	Flexural	Compressive	
Karsli et al. (2013) [52]	ABS	Glass fiber	Fiber content: 5–40 wt%	ISO 527	-	-	UTS: ~87 MPa at 40 wt% fiber, 117% higher than pure ABS, Elongation decreased from 220 to 10%.
Tekinalp et al. (2014) [18]	ABS	Carbon fiber	Fiber content: 10, 20,30, 40 wt%	ASTM D638	-	-	UTS: 65 MPa at 40 wt% fiber, 85% higher than pure ABS. Tensile modulus 13.7 GPa at 30 wt% fiber, 585% higher than pure ABS.
Perez et al. (2014) [51]	ABS	Jute fiber	Fiber content: 5 wt%	ASTM D638	-	-	UTS: 25.9 MPa, 10% lower than pure ABS, fracture strength increased by 28%.
Carneiro et al. (2015) [53]	PP (Polypropylene)	Glass fiber	Fiber content: 30 wt%, with the variation of raster angle	DIN 53504	-	-	Tensile modulus and strength increased by 30% and 40%, respectively, compared to pure PP.
Ning et al. (2015) [28]	ABS	Carbon fiber	Fiber content: 3, 5, 7.5, 10, and 15 wt%, with fiber length 0.1 and 0.15 mm	ASTM D638	ASTM D790	-	UTS 42 MPa at 5wt% fiber, 20% higher than pure ABS; Lowest UTS 34 MPa at 10 wt% fiber, 2.85% lower than pure ABS. Young’s modulus 2.5 GPa at 7.5 wt% fiber, 30% higher than pure ABS; ductility decreased as fiber content increased. Flexural strength, flexural modulus, and flexural toughness increased by 11.82%, 16.82%, and 21.86%, respectively, compared to pure ABS, at 5wt% fiber.
Dul et al. (2016) [20]	ABS	Graphene (xGnP)	Fiber content: 2, 4, and 8 wt%	ISO 527	-	-	UTS decreased as xGnP content increased, lowest at UTS with 8 wt% of xGnP, 7% lower than pure ABS.
Halápi et al. (2018) [54]	PLA	Glass fiber	Fiber content: 15 wt%	ISO 3167	-	-	UTS: 45.38 MPa, 2.2% higher than pure PLA.

**Table 3:** Research on Continuous Fiber Reinforcement.

Authors	Materials		Methods	Dimensions & Testing Standards			Outcomes
	Base	Addition		Tensile	Flexural	Compressive	
Li et al. (2016) [57]	PLA	Carbon fiber	Treatment with methylene dichloride solution for both PLA and carbon fiber (Modified CCFR)	Not Standardized	Not Standardized	-	UTS: 91 MPa, 13.75% higher than the material with CCFR (continuous carbon fiber reinforcement), and 225% higher than pure PLA. FS: 156 MPa, 164% higher than the material with CCFR, and 194% higher than pure PLA.
Matsuzaki et al. (2016) [63]	PLA	Carbon and Jute fiber	Vol fraction of 6.6% and 6.1% for carbon and jute, respectively	JIS K 7162 for jute. Carbon not standardized	-	-	Carbon > Jute UTS and modulus carbon-reinforced are 185.2 ± 24.6 MPa and 19.5 ± 2.08 GPa, respectively, which are 435% and 599% higher than those of the pure PLA for UTS and modulus, respectively. Failure mode was brittle.
Tian et al. (2016) [60]	PLA	Carbon fiber	1000 fibers in a bundle, variation of printing parameters	-	ISO 14125	-	The strength and modulus increased with increasing extrusion temperature; the maximum strength and modulus were 155 MPa and 8.6 GPa, respectively, at 240 °C. The strength and modulus decreased with increased layer thickness and hatch spacing. The printing speed did not have a significant effect on strength and modulus.
Li et al. (2019) [64]	PLA	Carbon fiber	Variation of fibers content of 1, 3, 5, 7, 10, 15 wt%	National Standard (China)	-	-	UTS increased with increasing fiber content; maximum UTS: 106.3 MPa, 178% higher than pure PLA, at 15 wt% fiber content.
Le Duigou et al. (2019) [61]	PLA	Flax fiber	Filament diameter 482 ± 30 μm. Raster angle of 0° and 90°	ISO 527	-	-	0° raster angle and 30 vol% fibers had the highest UTS: 253.7 ± 15 MPa, improved by 4.5× in terms of strength and 7× in terms of stiffness compared to pure PLA.
Mangat et al. (2018) [65]	PLA	Silk and Sheep Wool	Chemical treatment for silk and sheep wool, final diameter ~11μm. Variations of printing parameters, and fibers insertion sequence	-	ASTM D790	-	FS: 24.58 MPa, using silk at 100% infill, 0°/90° raster angle, and 4 laminates; 52% lower than pure PLA.
Heidari-Rarani et al. (2019) [59]	PLA	Carbon fiber	Carbon fiber diameter 7μm. Chemical treatment for carbon fiber and extruded, the final diameter of the fiber is 1 mm	ASTM D638; ASTM D3039	ASTM D790	-	UTS and modulus increased by 36% and 208%, compared to pure PLA; Failure strain decreased by 62%. FS and modulus increased by 109% and 367% compared to pure PLA.
Naranjo-Lozada et al. (2019) [66]	Nylon	Carbon fiber	Variation of volume fraction of fiber and fiber placement arrangement	ASTM D638	-	-	UTS: 304.3 MPa at 54 vol% fiber which was 25× higher than pure nylon and reached an elastic modulus of 23 GPa. The wider arrangement showed slightly better performance than the thinner one.
Dickson et al. (2017) [62]	Nylon	Carbon, Glass, and Kevlar fiber	Variation of raster pattern (Concentric and Isotropic); Fiber bundle diameter: 8 μm for carbon, 12 μm for kevlar, and 10 μm for glass	ASTM D3039	ASTM D790	-	Carbon > Glass > Kevlar The isotropic pattern was better than the concentric pattern. UTS: 216 MPa with carbon fiber, 254% higher than pure nylon. The failure mode was brittle. FS: 250.23 MPa with carbon fiber, 496% higher than pure nylon. As the fiber volume increased, both tensile and flexural strengths also increased.

**Table 4:** Research on Powder Addition Reinforcement.

Authors	Materials		Methods	Dimensions & Testing Standards			Outcomes
	Base	Addition		Tensile	Flexural	Compressive	
Masood and Song (2004) [68]	Nylon	Fe	Powder content of 40 vol% fine (<30 μm) and coarse (50–80 μm), and 30 vol% coarse.	Filament testing	-	-	Highest UTS, Tensile modulus, and tensile strain at break were 3.87 MPa, 54.52 MPa, and 16.82%, respectively at 30% Fe (Coarse).
Nikzad et al. (2011) [69]	ABS	Fe	Powder content of 5, 10, 20, 30, and 40 vol%	Not Standardized	-	-	Iron-filled ABS had characteristics of brittle and hard material with much lower elongation. Tensile strength drops significantly (25%) as a result of the addition of 10 vol% of iron powder
Karsli et al. (2013) [52]	ABS/PA6	CaCo3	Powder content of 5–30 wt%,	ISO 527	-	-	UTS increased by 15% with 5 wt% addition of CaCo3 powder and then decreased as the powder content increased. Elongation decreased from 220% to 62%
Perez et al. (2014) [51]	ABS	TiO2	Powder content of 5 wt%	ASTM D638	-	-	The UTS increased by 12.98% compared to pure ABS, but the strain decreased by 10%.
Weng et al. (2016) [71]	ABS	OMMT	Powder content of 1, 3, and 5 wt%	ASTM D638	ASTM D790	-	Tensile strength and elastic modulus increased by 43% and 200%, respectively, Flexural strength increased by 33.3%.
Osman and Atia (2018) [72]	ABS	RS	Powder content of 5, 10, and 15 wt%, variation of raster angle	ASTM D638	ASTM D790	-	UTS decreased as the RS content increased, then increased again until reaching maximum UTS at 15 wt% of RS content at a 0° raster angle. The tensile modulus decreased as the RS content increased. FS decreased as the RS content increased, then increased again until reaching maximum FS at 15 wt% of RS content at a 0° raster angle. The flexural modulus decreased as the RS content increased, and then increased again until reaching a maximum modulus at 15 wt% of RS content at a 0° raster angle.
Çanti and Aydin (2018) [67]	ABS	Al & ZrB2	Powder content of 1.5 wt%	ISO 527	ASTM D790	-	UTS increased by 0.3% and 12.6% with the addition of Al and ZrB2, respectively. The strain increased by 85% and 108% with the addition of ZrB2 and Al, respectively. FS decreased around by 5% with the addition of 1.5 wt% Al. FS increased by 8.7% with the addition of ZrB2. The deflection property improved by 3.7 and 26 percent with Al and ZrB2 addition, respectively.
Ecker et al. (2019) [73]	PLA	Wood	Powder content of 15 and 30 wt%	ASTM D638	-	-	UTS decreased as the wood powder content increased, whereas the water absorption increased as the wood powder content increased.
Sezer and Eren (2019) [74]	ABS	MWCNT	Powder content of 1, 3, 5, 7, and 10 wt%, variation of raster angle	ASTM D412	-	-	UTS was remarkably increased by 288% compared to pure ABS at 7 wt% of MWCNT at a 0°/90° criss-cross raster angle.
Walker et al. (2020) [70]	PLA	AgSMW (Silver sub-micron)	Powder content of 0.1, 1, and 10 wt%	ASTM D638	-	-	UTS and strain at break decreased moderately as the AgSMW content increased, whereas the tensile modulus did not chang significantly. However, the addition of 10 wt% of AgSMW significantly reduced bacteria growth by close to 50%.

**Table 5:** Research on Vibration-Assisted FFF.

Authors	Materials		Methods	Dimensions & Testing Standards			Outcomes
	Base	Addition		Tensile	Flexural	Compressive	
Keleş et al. (2018) [76]	ABS	Carbon fiber	Vibration on printing head. Amplitude ~0.15 mm, wavelength 4.8 mm at ~375 Hz.	Not Standardized	-	-	Total porosity decreased from 13 to 10 vol%. The fracture strength, tensile strength, and nominal strain-at-break increased by more than 10% compared to non-vibrated specimens. Elastic modulus improved from 2.5 ± 0.1 GPa to 2.7 ± 0.1 GPa.
Jiang et al. (2020) [77]	PLA	-	Vibration on printing head. A vertical direction of vibration at 100 Hz, an amplitude of 0.35 g. Variation of build orientation.	ISO 527	-	-	Tensile strength increased by almost 50% at Z orientation. At X orientation, the tensile strength was not much different to non-vibrated specimens.

**Table 6:** Research on annealing post-processing.

Authors	Materials		Methods	Dimensions & Testing Standards			Outcomes
	Base	Addition		Tensile	Flexural	Compressive	
Jo et al. (2018) [81]	PLA	-	Variation of annealing time and temperature. Variation of applied pressure.	ASTM D638	-	-	UTS of specimens that were reheated at 160 °C for 30s increased by 5% compared to non-reheated samples. A combination of a reheating process for 120 s and an applied pressure of 19 N resulted in the improvement of UTS by 10.2% more than the reheating process alone.
Behzadna-sab et al. (2019) [82]	PLA	-	Variation of annealing time and temperature.	ISO 527	-	-	UTS decreased with the increase of annealing temperature or time, with no significant change in the modulus. This is mainly due to the degradation process of PLA molecules.

**Table 7:** The advantages and disadvantages of each reinforcement method.

Methods	Advantages	Disadvantages
Short Fiber Reinforcement (SFR)	i. Simple, as it is based on a filament reinforcement method; ii. Easy to control filler contents during the filament extrusion process.	i. Inconsistent filament size; ii. Nozzle clogging. iii. Non-uniform volume fraction and sizes of the particle over the polymer matrix.
Continuous Fiber Reinforcement (CFR)	i. The extrusion process is not required; ii. The printed parts have uniform properties.	i. The extruder and nozzle modifications are required. ii. Fiber breakage during the printing process.
Powder Addition Reinforcement (PAR)	i. Simple, as it is based on a filament reinforcement method; ii. Easy to control filler contents during the filament extrusion process.	i. Inconsistent filament size; ii. Nozzle clogging; iii. Non-uniform volume fraction and sizes of the particle over the polymer matrix.
Vibration-Assisted FFF	i. Easy to control without having to change G-Code used for the printing process; ii. No special equipment is required.	i. The stepper motors used in FFF printers are easily damaged; ii. Wavy and inconsistent printed parts; iii. The dimensions of the printed part could be slightly distorted due to the design.
Annealing	i. A simple post-processing technique; ii. Uniform cooling.	i. Changes in the chemical and thermal properties of printed parts are possible.
